# Supplementary material for: Implementation and benchmarking of a novel analytical framework to clinically evaluate tumor-specific fluorescent tracers
Source: Nat Commun. 2018 Sep 18;9:3739. doi: 10.1038/s41467-018-05727-y (PMC6143516; doi:10.1038/s41467-018-05727-y)
Supplement: Supplementary file 1 — Supplementary Information [file 41467_2018_5727_MOESM1_ESM.pdf]

Supplementary information

Implementation and Benchmarking of a Novel Analytical Framework for the Clinical Evaluation of Tumor-specific Fluorescent Tracers

Koller et al.

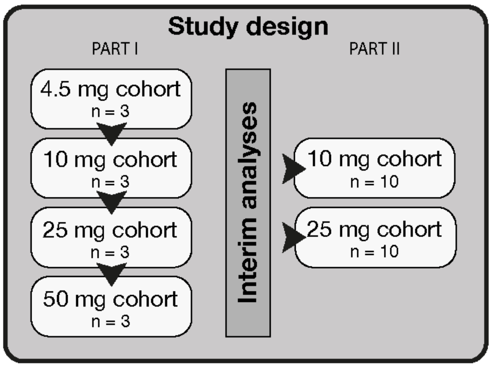

Supplementary Figure 1 - Flow chart of the study design. In part I, four dose groups of three patients each were included and evaluated. In part II, the two best performing dose groups were expanded to a total of 10 patients each.

Supplementary Figure 2 – page 1/4

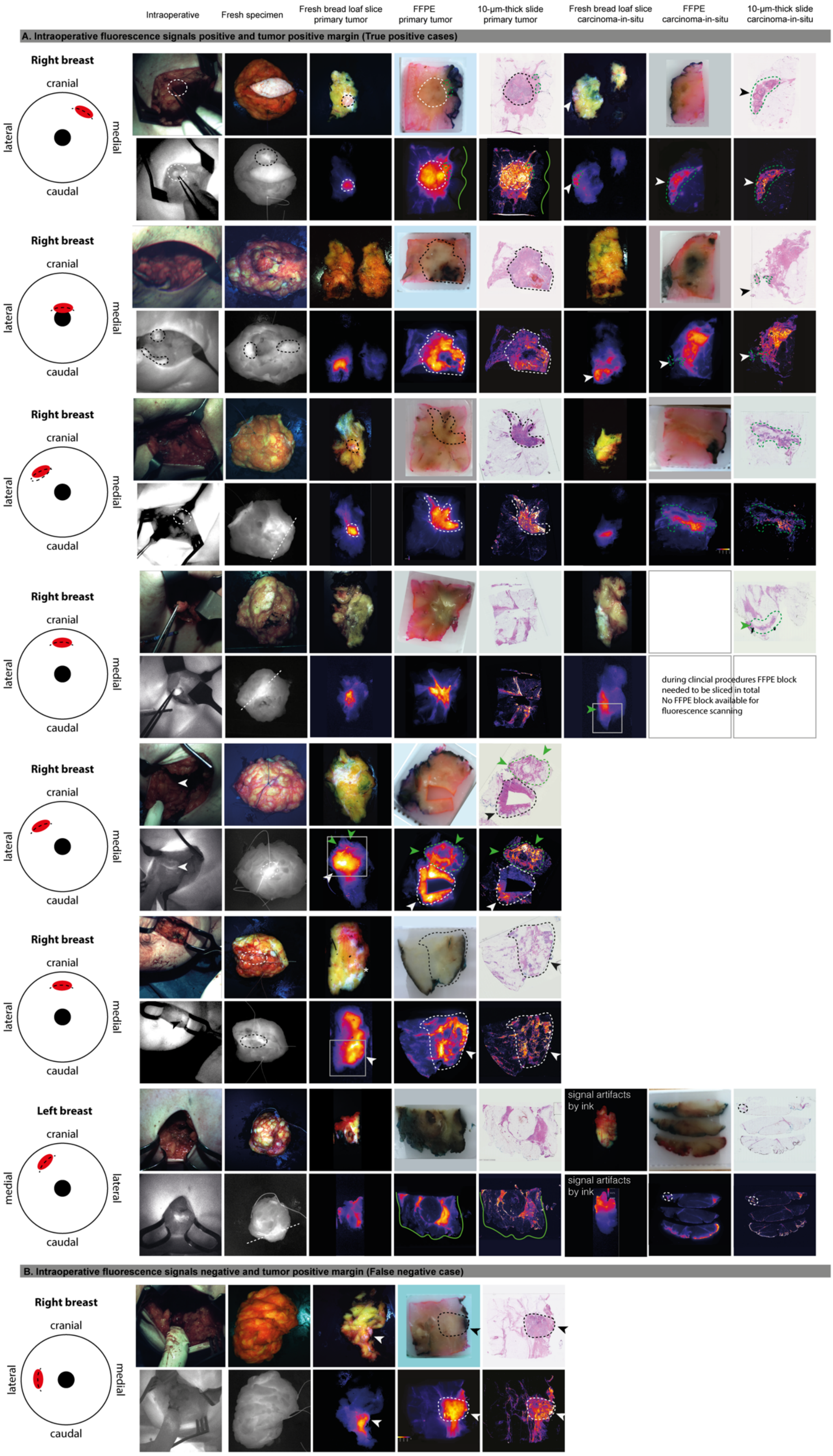

Supplementary Figure 2 – continued page 2/4

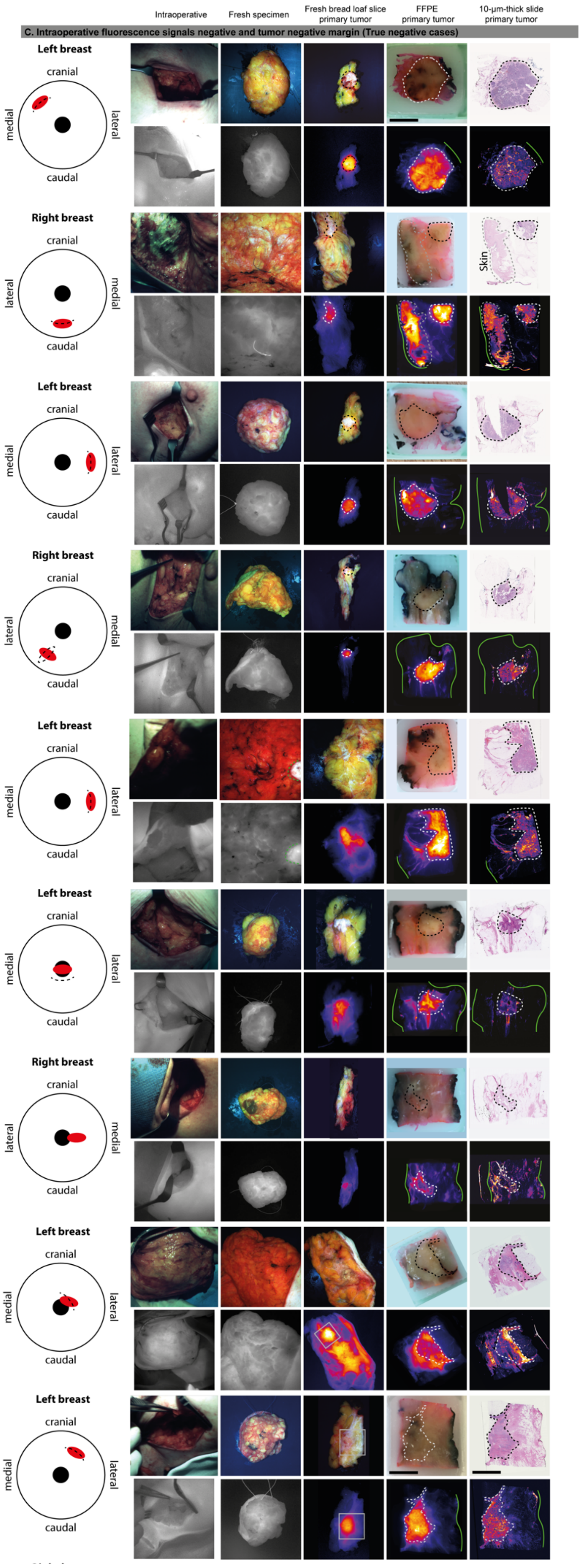

Supplementary Figure 2 – continued page 3/4

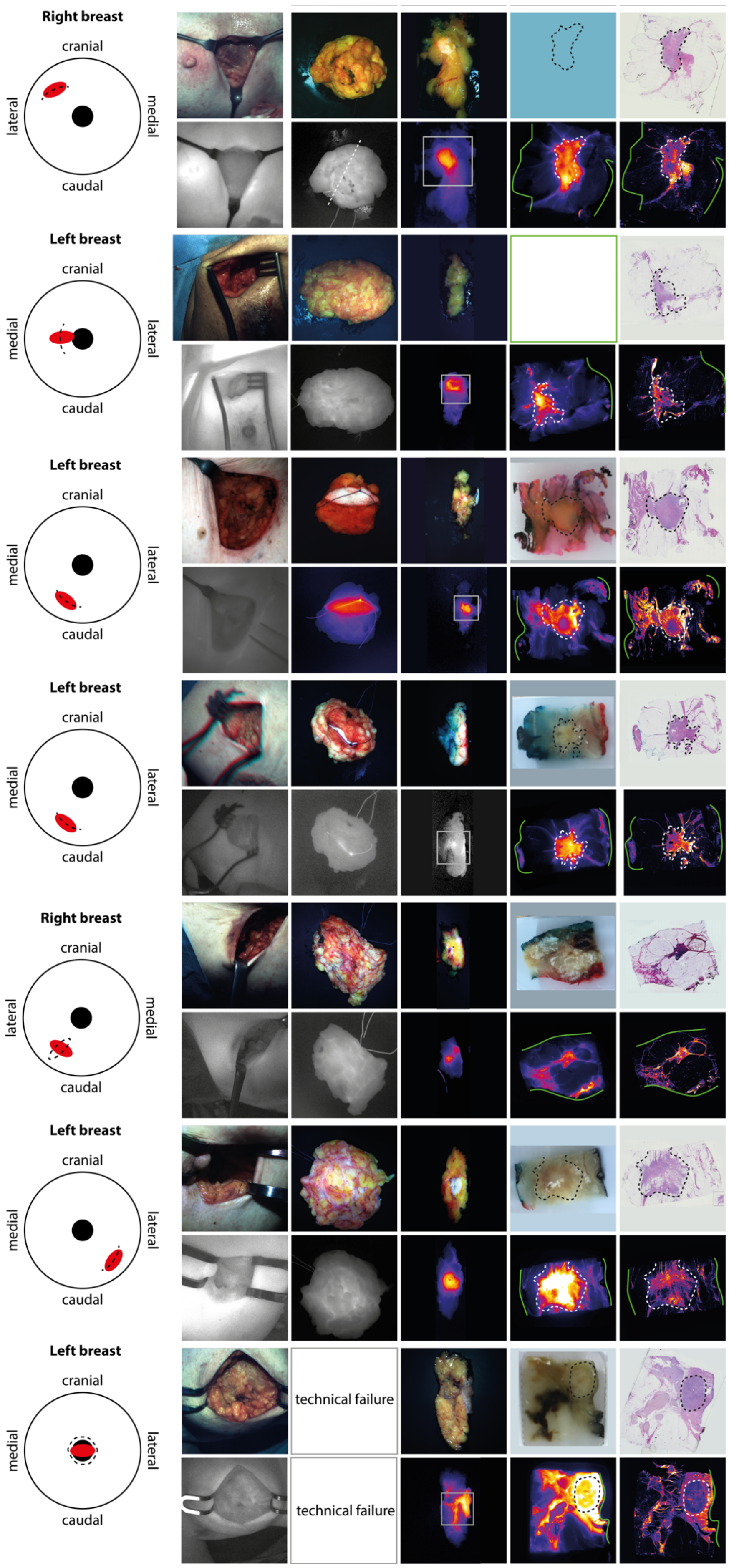

Supplementary Figure 2 – continued page 4/4

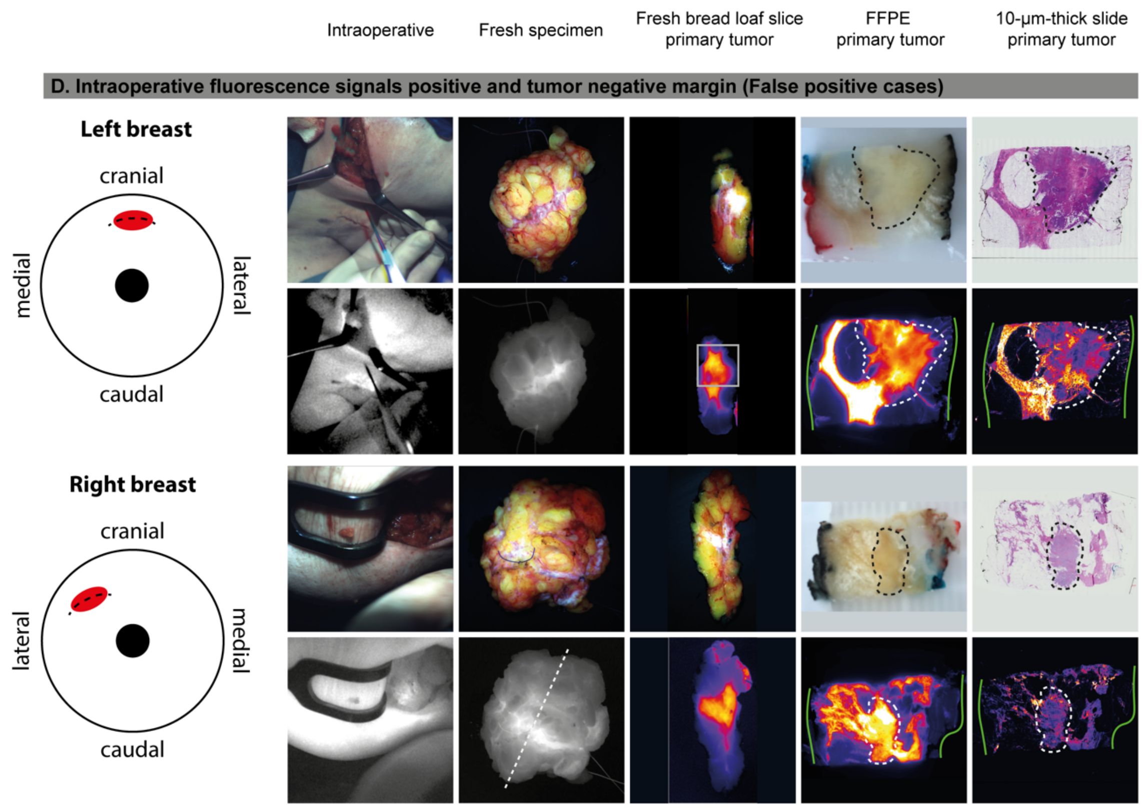

Supplementary Figure 2 – Overview of imaging data of all patients with all imaging modalities. Columns from left to right represent: the location of the tumor in the breast, intraoperative images, images of the fresh specimens, images of the fresh tissue slices, images of FFPE blocks of the primary tumor, and images of the 10-μm-thick sections of the primary tumor. When the surgical margin of carcinoma in situ components were positive these were added in the additional columns. Patients with positive fluorescence signals in the cavity and a histologically proven tumor positive surgical margin are presented in (**a**). Patients with negative fluorescence signals in the cavity and a histologically proven tumor positive surgical margin are presented in (**b**). Patients with negative fluorescence signals in the cavity and a histologically proven tumor negative surgical margin are presented in (**c**). Patients with positive fluorescence signals in the cavity and a histologically proven tumor negative surgical margin are presented in (**d**). *Tumors are delineated with a dashed line. Location of a tumor positive surgical margin is indicated with an arrow. The inked margin is indicated with a green line in the fluorescence images of the FFPE blocks and 10-μm-thick sections.*

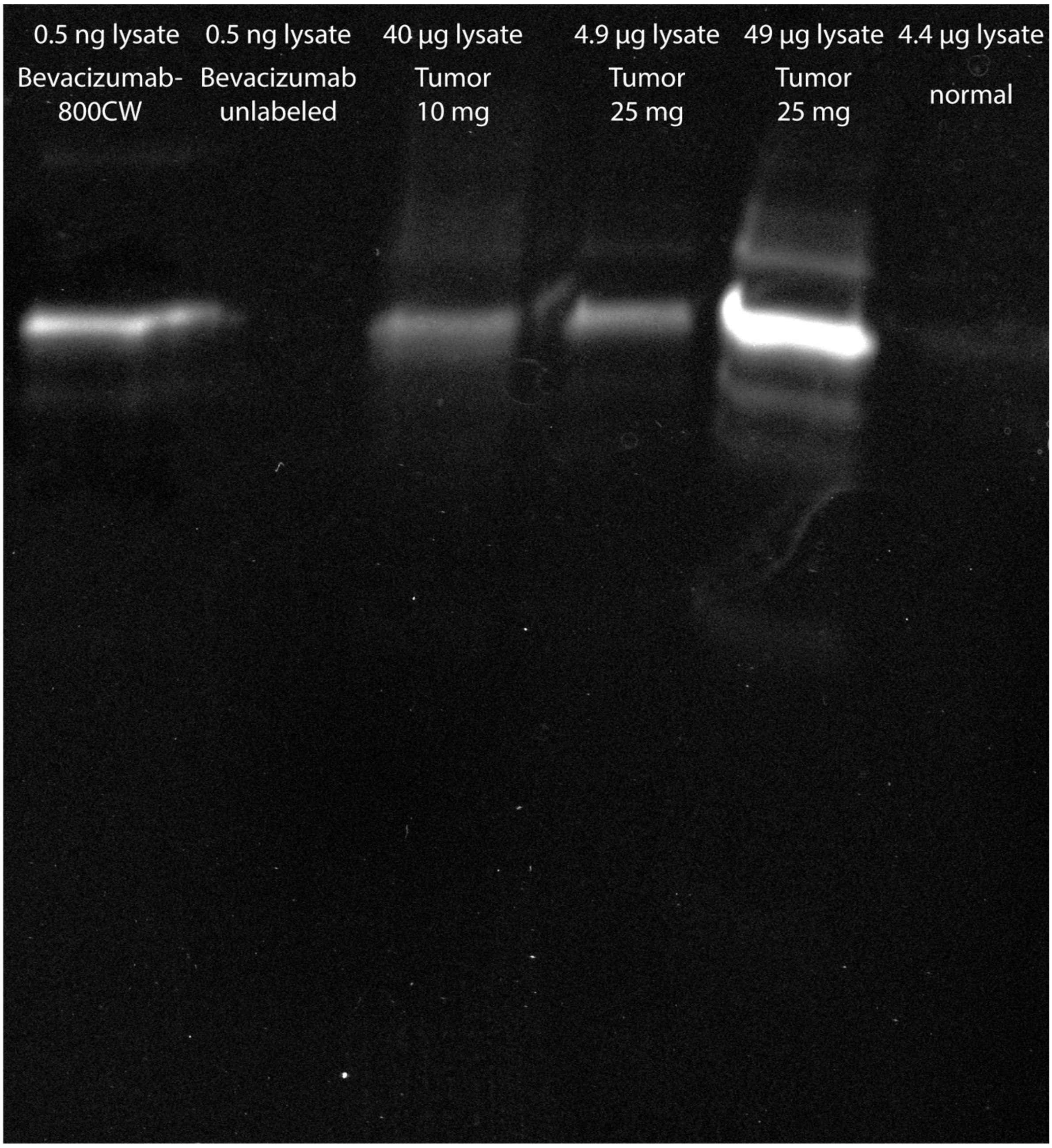

Supplementary Figure 3 – SDS-PAGE from tumor lysates and normal tissue lysates compared with diluted bevacizumab-800CW to determine the intactness of the tracer in human tissue. Bands from left to right: diluted bevacizumab-800CW, unlabeled bevacizumab, tumor lysate of a patient from the 10mg dose group, tumor lysate of a patient from the 25mg dose group, tumor lysate of the same patient from the 25mg dose group but with a comparable lysate weight with the 10mg patient, lysate of normal tissue. The bands of the human tissue lysates is present at the same hight as the diluted bevacizumab-800CW, which indicates the intactness of the tracer in human tissue.

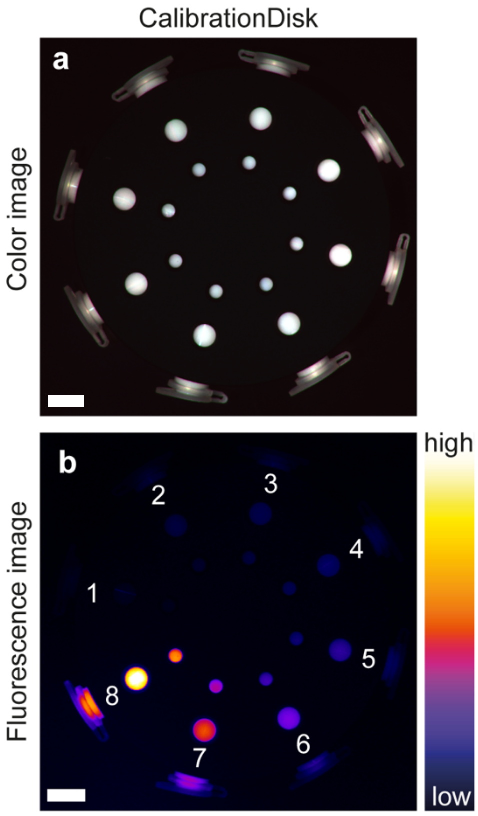

Supplementary Figure 4 – System calibration using the CalibrationDisk. Before and after each surgical procedure the intraoperative camera system and the light-tight macroscopic imaging device were calibrated using a calibration device (CalibrationDisk, SurgVision BV, The Netherlands). The device consists of a disk with round windows that can hold 8 clear polypropylene tubes of 0,65 ml, that were filled with 2% intralipid and two-fold increasing concentrations of bevacizumab-800CW: in tube 1 no tracer was added, in tube 2 a concentration of 1:100, in tube 3 of 1:200, in tube 4 of 1:400, in tube 5 of 1:800, in tube 6 1:1600, in tube 7 1:3200 in tube 8 of 1:6400. a color image of the CalibrationDisk is depicted in image **a**, and the corresponding fluorescence image is depicted in **b**. Scale bar represent 1 cm.

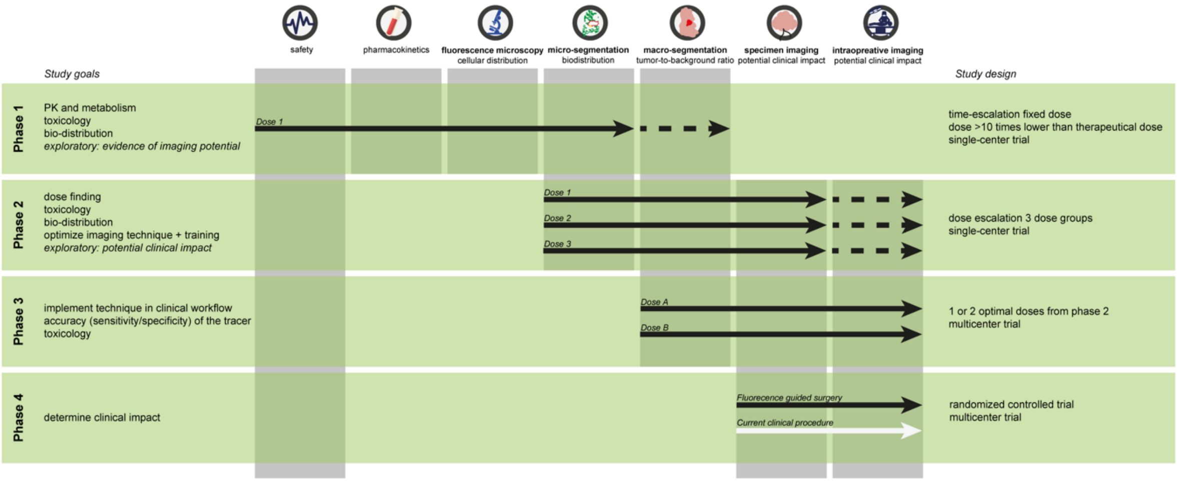

Supplementary Figure 5 – Platform for fluorescent tracer evaluation in clinical trials. The horizontal rows depict the proposal for clinical trial design from phase I until phase IV clinical trials. In the vertical columns, the analytical methods that need to be performed to reach the study goals are depicted. Arrows represent conclusions that can be made from each analysis. Dashed arrows represent exploratory data analyses to determine the potential clinical application.
